# Synaptopathies in Developmental and Epileptic Encephalopathies: A Focus on Pre-synaptic Dysfunction

**DOI:** 10.3389/fneur.2022.826211

**Published:** 2022-03-08

**Authors:** Giulia Spoto, Giulia Valentini, Maria Concetta Saia, Ambra Butera, Greta Amore, Vincenzo Salpietro, Antonio Gennaro Nicotera, Gabriella Di Rosa

**Affiliations:** ^1^Unit of Child Neurology and Psychiatry, Department of Human Pathology of the Adult and Developmental Age “Gaetano Barresi”, University of Messina, Messina, Italy; ^2^Department of Neuromuscular Disorders, Institute of Neurology, University College London, London, United Kingdom; ^3^Pediatric Neurology and Muscular Diseases Unit, Istituto di Ricovero e Cura a Carattere Scientifico (IRCCS) Giannina Gaslini, Genoa, Italy; ^4^Department of Neurosciences, Rehabilitation, Ophthalmology, Genetics, Maternal and Child Health, University of Genoa, Genoa, Italy

**Keywords:** synaptopathy, developmental and epileptic encephalopathy (DEE), pre-synaptic mechanisms, drug resistant epilepsy, intellectual disability (ID), SNAREopathies

## Abstract

The proper connection between the pre- and post-synaptic nervous cells depends on any element constituting the synapse: the pre- and post-synaptic membranes, the synaptic cleft, and the surrounding glial cells and extracellular matrix. An alteration of the mechanisms regulating the physiological synergy among these synaptic components is defined as “synaptopathy.” Mutations in the genes encoding for proteins involved in neuronal transmission are associated with several neuropsychiatric disorders, but only some of them are associated with Developmental and Epileptic Encephalopathies (DEEs). These conditions include a heterogeneous group of epilepsy syndromes associated with cognitive disturbances/intellectual disability, autistic features, and movement disorders. This review aims to elucidate the pathogenesis of these conditions, focusing on mechanisms affecting the neuronal pre-synaptic terminal and its role in the onset of DEEs, including potential therapeutic approaches.

## Introduction

**Figure 1:**
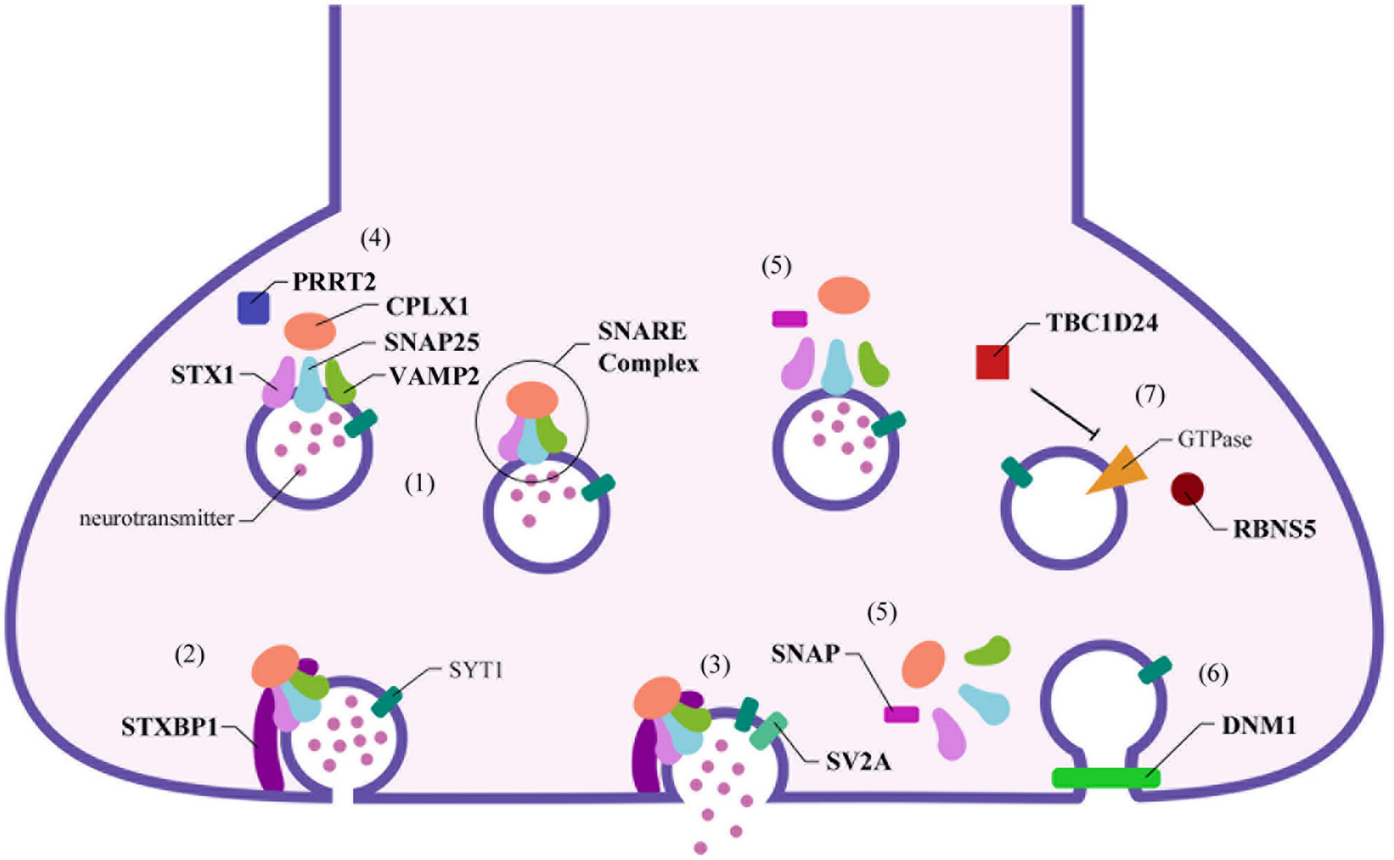
Schematic representation of pre-synaptic proteins localization and their role in the synaptic functioning. Numerous proteins interact together and with the cellular membrane on the pre-synaptic terminal to release the neurotransmitters in the synaptic cleft. (1) Stx1, Snap25, and Vamp2 form the core of the SNARE complex, which is stabilized by Cplx1. (2) When the synaptic vesicle reaches the cellular membrane, Stxbp1 binds Stx1, starting the priming and the rapid fusion of the vesicle. (3) Sv2a, the only specific synaptic protein, interacts with Syt1 and induces neurotransmitter release. (4) On the contrary, Prrt2 inhibits this process by reducing the formation of the SNARE complex. (5) The disassembly of the SNARE complex is mediated by Snap proteins, (6) allowing the subsequent clathrin-mediated endocytosis, with the participation of the Dnm1. (7) This process is coordinated by several GTPases: Tbc1d24 regulates Rab35, while Rbns5 interacts with Rab4 and Rab5 to lead the empty synaptic vesicle toward the endosome for recycling.

Optimal synaptic communication is a complex and finely regulated process that is fundamental for proper nervous system physiology (1–3). The expanding knowledge in the neurobiological field has allowed to increase the accuracy of the etiopathogenesis definition of nervous system diseases: from the macroscopic involvement of anatomical structures and circuitry, the focus shifted to the microscopical elements of this system, including subcellular ones, as transporting proteins, signaling superficial molecules, receptors, and neurotransmitters. The synapse plays a central role in this exchange of information and represents the essential signal transmitting unit of the nervous system (1, 3). The neurotransmitters release requires the availability of synaptic vesicles, which undergo immediate fusion with the pre-synaptic membrane when the action potential arrives (4). The synaptic vesicles undergo repeated recycling, and this process involves the sequential participation of several proteins (see Figure 1) (2, 5).

A synaptopathy is defined as an alteration in the functionality of any element constituting the synapse: the pre- and post-synaptic terminals, the synaptic cleft, and all the surrounding components, such as glial cells and extracellular matrix (1, 3, 6). Although the first reference to the term “synaptopathy” was made in 2003 by Li et al. regarding the Huntington Disease (7), in the last few decades, pathogenic variants in genes encoding synaptic proteins have been demonstrated to determine altered protein levels/function in several neuropsychiatric diseases, such as epilepsy, intellectual disability (ID), and autism spectrum disorder (1, 8).

The term “epileptic encephalopathy” describes a catastrophic form of epilepsy, with a frequent onset in infancy or early childhood, in which the epileptic activity itself significantly contributes to severe developmental delay (DD) and behavioral impairments (9, 10). In those patients presenting with pre-existing DD, the effect of the epileptic activity causes a worsening of the developmental consequences arising directly from the genetic mutation, configuring a clinical phenotype called “developmental and epileptic encephalopathy” (DEE) (10).

Since 2001, when a genetic cause for an epileptic encephalopathy was first reported (11), numerous genes have been associated with DEEs, and several synaptopathies have been described (9). Given that numerous proteins participate in the mechanisms underlying the correct functioning of the synapse, these disorders are generally studied centering the attention on the single affected gene (2). We focused our research on those genes involved in the pathogenesis of DEEs affecting the pre-synaptic compartment, consisting of the axon terminal and the proteins implicated in releasing neurotransmitters. Since these genes are often identified in the context of large cohorts of patients tested with genetic panels, an accurate description is not always available, making it challenging to characterize clinical phenotypes and to identify the DEEs. Therefore, we considered all patients presenting with epilepsy and intellectual disability (ID).

## STX1B

Syntaxin1 (Stx1) is a protein widely expressed in the nervous system (12, 13) and, together with Snap25 (encoded by *SNAP25*) and synaptobrevin-2 (encoded by *VAMP*2), form a stable complex called soluble N-ethylmaleimide-sensitive factor (NSF) attachment protein receptor (SNARE) complex, made by a four-helix bundle implicated in Ca^2+^-dependent exocytosis of the synaptic vesicles and neurotransmitter release (14, 15). Vamp2 protein represents the vesicle membrane portion of SNARE neuronal complex (v-SNARE), while the plasma membrane of SNARE (t-SNARE) is constituted by Stx1a and Snap25 (16).

Stx1 is an integral membrane protein composed of three functional domains: (1) the N-terminal peptide, (2) an α-helical domain which is called Habc domain, and (3) the SNARE and transmembrane motif in the C-terminal region (13, 17–20). There are two different protein configurations, namely the “closed” and the “open” Stx1. The first one is characterized by a link between the Habc domain and the N-peptide. Switching from the closed to the open conformation is crucial in regulating the exocytosis mediated by the SNARE complex, and Stx1 plays a crucial role in the initiation of synaptic exocytosis.

The first step that allows the SNARE complex assembly is represented by the binding between Munc18 (encoded by *STXBP1*) and Stx1 in its closed form.

This is the starting point of a process that will end with releasing the neurotransmitter in the synaptic cleft (21–23).

There are two isoforms of Stx1 called 1a and 1b. Even if they share 84% of their amino acid sequence and the basic function as neuronal t-SNAREs, Stx1b is the principal mediator for spontaneous and evoked fast synaptic vesicle exocytosis (24, 25).

The gene encoding for Stx1a is located on chromosome 7 and is one of the genes involved in Williams-Beuren Syndrome (WBS), caused by deletion 7q11.23 (26). In this regard, Gao et al. (27) showed that the level of expression of Stx1a is correlated with the degree of intelligence in patients with WBS. It has also been found that some variants of *STX1A* are associated with an increased chance of developing migraines (28). Moreover, data from studies conducted on many families investigated with Whole Exome Sequencing (WES) suggest that *STX1A* may be a possible candidate gene in the development of neurodevelopmental disorders (29).

On the contrary, less is known about the involvement of pathogenic variants of *STX1A* in the development of epilepsy. However, the influence that Stx1a exerts on glutamate uptake and glutamatergic transmission could partially explain the role of *STX1A* in epileptogenesis. Data from the literature suggest that Stx1a acts by enhancing the internalization of excitatory amino acid transporter 1 (EAAC1) responsible for glutamate re-uptake: increased internalization and reduced expression on the cell surface cause an overall reduction in glutamate uptake (30). Additionally, a Stx1a role in regulating voltage-gated K+ channel determined by physical interaction of this protein with the ion channels has been demonstrated in animal models (31). Moreover, single nucleotide polymorphisms (SNP) of *STX1A* and *VAMP2* have been described in association with cryptogenic epilepsy (32).

Much more has been described regarding the 1b isoform. *STX1B* gene is located on chromosome 16 (16p11.2 region) (33) and studies conducted on mouse models have shown an early death of the animals and an altered function of the neuromuscular junction in KO mice for *STX1B* (34). These findings underline the critical role played by Stx1b in the proper signaling of the nervous system. Therefore, dysfunctions of this protein, whether due to mutations or deletions, are associated with the development of various disorders of the nervous system, including ID, speech disorders, and various forms of epilepsy (35).

To the best of our knowledge, *STX1A*-related DEEs are not reported in the literature. For this reason, we focused on the 1b isoform and identified 20 patients (35–39). Clinical information was collected about the age onset of symptoms, the type of seizures, the presence of febrile seizures (FS), electroencephalographic patterns, and the development of ID and/or movement disorders. Not all clinical information could be traced for all patients.

The age of onset of epilepsy ranged from a few days after birth to a maximum of 4 years (35–39). Most of them present multiple forms of epilepsy: the most represented type of seizures are myoclonic ones (11/20), generalized tonic-clonic ones (10/20), and absences (9/20). Other types of seizures reported are atonic (7/20), tonic (6/20), focal (3/20), spasms (1/20), myoclonic-astatic epilepsy (1/20). FS were also frequent, reported in seven (7/20) patients. The increased susceptibility to develop FS has been reported in previous studies (35, 40, 41), and more recently by Mishima et al. (42). In this cohort of patients, we report a recurrence of ataxia, present in 11 of 20 cases (35, 37, 39).

In addition, seizure control was reached in five patients, but given the large number of drugs administered, often in co-administration, it is difficult to define the most appropriate pharmacological treatment. Clinical data are summarized in Table 1.

**Table 1:** Clinical features of *STX1B*-related patients.

**No**.	**Age of onset**	**Seizure type at onset**	**Epilepsy evolution**	**Eeg**	**Movement disorders**	**ID/DD**	**Genetic variant**	**Reference**
1	1 years	GTCS, FS	MAE	High-amplitude polyspike waves	NA	Moderate to severe ID	Arr[hg19] 16p11.2 (30, 943, 951–32, 151, 753) x1 (*de novo*)	(38)
2	NA	NA	NA	NA	NA	NA	c.140C>A; p.s47* (heterozygous variants)	(35)
3	3 years and 6 months	GTCS, TS, Myo, Abs	NA	Focal sharp wave, generalized sharp wave	Ataxia	Moderate ID	c.657T>A; p.Val216Glu	(35)
4	20 months	GTCS, Myo, Abs, AS, TS	NA	Generalized sharp wave, focal sharp wave	Ataxia	DD	c.676G>C; p.Gly226Arg	(35)
5	13 months	Myo, ats, GTCS	NA	focal sharp wave	Ataxia	DD	Arr[hg19] 16p11.2 (30, 332, 532–31, 104, 012) x1	(35)
6	9 months	Myo, Atyp Abs, GTCS, TS	NA	Generalized sharp wave, focal sharp wave	Ataxia	DD	c.563dupA; p.Asn189Alafs*5	(35)
7	16 months	Myo, Atyp Abs, GTCS, TS	NA	Generalized sharp wave, focal sharp wave	Dystonia	DD	c.563dupA; p.Asn189Alafs*5	(35)
8	2 years	Abs, Myo, as, TS	NA	Generalized polispike sharp wave, generalized sharp wave	Ataxia	DD	c.845T>C; p.Ile282Thr	(35)
9	NA	NA	NA	NA	NA	NA	c.773G>A; p.Ser258Asn	(35)
10	3 years	GTCS, Abs, Myo, AS, FS	NA	Generalized polispike sharp wave, gps	Mild ataxia	DD	c.662T>C; p.leu221Pro	(35)
11	4 years	GTCS, Myo, Abs, AS	NA	Generalized sharp wave	Ataxia	DD	c.155delA; p.q52rfs*2	(35)
12	10 months	Abs	NA	NA	Ataxia, tremor, dysarthria	DD	c.431G>T; p.Cys144Phe	(35)
13	Since birth	IS	NA	Hypsarrhythmia	NA	Severe ID	c.736 G>C; p.Ala246Pro	(35)
14	2 years	AS, Abs, Myo, GTCS	NA	Generalized polispike sharp wave, generalized sharp wave	Ataxia	ID	c. (?_242)_(*3565_?)	(35)
15	2 years	AS, Abs, Myo, GTCS	NA	Generalized polispike sharp wave, generalized sharp wave	Ataxia	ID	c. (?_242)_(*3565_?)	(35)
16	3 months	Myo, apnea and cyanosis	NA	Generalized polispike sharp wave	NA	DD	c.383del; p. Gln128Glyfs*2 (heterozygous variant)	(35)
17	NA	AS, GTCS	NA	Focal sharp wave	/	Mild ID	c.420C>G; p. Tyr140*	(35)
18	13 months	TC, MA, myo, and TS	MAE	Generalized epileptic activity	Ataxia and tremor	Moderate ID	c.676G>C; p.Gly226Arg	(39)
19	NA	Dravet-like	NA	NA	NA	ID	NA	(36)
20	9 months	FS	NA	Focal onset seizure disorder of temporal origin.	Cerebellar ataxia	Mild ID	Deletion of the full coding sequence of stx1b	(37)

## SNAP25

The *SNAP25* gene is mapped on chromosome 20 (20p12.2 region), and it encodes the synaptosomal-associated protein 25 kDa (Snap25) highly expressed in nerve and neuro-endocrine cells. Snap25 is a crucial component of the SNARE complex and, together with Stx1 and synaptobrevin-2, plays a crucial role in Ca^2+^-dependent exocytosis of the synaptic vesicles (14).

In mammals, due to the differential splicing of the *SNAP25* gene, two different isoforms (Snap25a and Snap25b) are obtained, which differ only for nine amino acids. However, they show different expression and localization profiles in the various brain regions in humans and mice (43). The most crucial isoform in synaptic transmission is Snap25b, which is mainly expressed in the synapses of the central nervous system and the peripheral motor endplates, and it regulates the exocytosis of neurotransmitters. Given its fundamental role in nervous transmission, *de novo SNAP25* variants are associated with various neurological disorders, such as epilepsy, movement disorders, and psychiatric conditions (39).

We reviewed the recent literature and found the description of 18 cases in which pathogenic variants of *SNAP25* are associated with DEEs (15, 44–47). All clinical data are summarized in Table 2.

**Table 2:** Clinical features of *SNAP25*-related patients.

**No**.	**Age of onset**	**Seizure type at onset**	**Epilepsy evolution**	**EEG**	**Movement disorders**	**ID**	**Genetic variant**	**Reference**
1	Since early childhood	fs	Drug resistant epilepsy	Generalized atypical polyspike and wave discharges and diffuse slowing of the background rhythm	Fatigable weakness, ataxic dysarthria, paretic and ataxic gait	Yes	c. 200 T>A; p.lle67Asn	(46)
2	18 months	gtcs, fS	DEE	Generalized spike-wave and continuous spike and wave during sleep	Not present	Yes (moderate)	c.496G>T; p.Asp166Tyr	(47)
3	5 months	gTCS, FS	Intractable severe static encephalopathy	Mild generalized slowing, 2–2.5 hz generalized spike-and-slow wave complexes.	Spastic quadriparesis	Yes	c.142G>T; p.Val48Phe	(45)
4	13 yo and 2 months	abs, gs	NA	NA	Muscular hypotonia	Yes, severe	c.118A>G; p.Lys40Glu	(44)
5	8 yo and 1 months	gtcs	NA	NA	Cerebellar ataxia, hand flapping, resting and intention tremor	Yes, mild	c.127G>C; p.Gly43Arg	(44)
6	Infant	gtcs	NA	NA	NA	Yes	c.520C>T; p.Gln174*	(44)
7	5 yo and 19 months	Is, ts	NA	NA	Muscolar hypotonia, spasticity	Yes	c.149T>C; p. (leu50ser)	(15)
8	19 yo and 5 months	NA	NA	NA	Muscolar hypotonia, ataxia, tremor dystonia	Yes (moderate)	c.127G>C; p.Gly43Arg	(15)
9	4 yo and 3 months	gs	NA	NA	Ataxia	Yes (moderate)	c.127G>C; p.Gly43Arg	(15)
10	6 weeks	gs, fs	NA	NA	NA	Yes (profound)	c.170T>G; p.Leu57Arg	(15)
11	8 yo and 3 months	gs, fs	NA	NA	NA	Yes (severe)	c.212T>C; p.Met71Thr	(15)
12	17 yo	gs, fs	NA	NA	NA	Yes (moderate)	c.497A>G; p.Asp166Gly	(15)
13	3 yo and 6 months	IS, gs	NA	NA	Muscolar hypotonia, ataxia, tremor dystonia	Yes (profound)	c.521A>C; p.Gln174Pro	(15)
14	14 yo	Is	NA	NA	NA	Yes (moderate)	c.575T>C; p.Ile192Thr	(15)
15	3 months	IS	NA	NA	Spasticity and muscolar hypotonia	Yes (severe)	c.593G>C; p.Arg198Pro	(15)
16	3 yo and 10 months	Gs, fs	NA	NA	Ataxia and muscolar hypotonia	Yes (moderate)	c.596C>T; p.Ala199Val	(15)
17	2 yo and 6 months	gs	NA	NA	Dystonia and muscolar hypotonia	Yes (mild)	c.114+2T>G	(15)
18	2 yo	IS	NA	NA	Muscolar hypotonia, spasticity	Yes (profound)	c.520C>T; p.Gln174*	(15)

Most of the patients (13/18) showed seizures onset during the early childhood (ranging from 3 months to 8 years of age); two cases (2/18) presented with neonatal-onset epilepsy, while only three patients (3/18) showed epilepsy after the first decade of life (from 13 to 19 years of age). The semiology of the seizures appears to be heterogeneous: although most patients manifested generalized seizures (12/18), also focal seizures (5/18) and epileptic spasms (4/18) have been reported. In one case the description of the crisis was unavailable. Noteworthy, all patients (18/18) developed an intractable severe encephalopathy with moderate to severe ID. When available (3/18), the EEG showed multifocal abnormalities or generalized spike-and-slow wave complex, with a variable response to antiepileptic drugs (AEDs). Half of the patients (9/18) showed a negative MRI, and aspecific neuroradiological anomalies were reported only in three cases (3/18), such as leukoencephalopathy or brain volume loss. Frequent association with movement disorders, such as tremors, dystonia, muscular hypotonia, spasticity, and cerebellar ataxia, is described (13/18), and only in one patient (1/18) the absence of movement disorders was reported. Three patients (3/18) also showed behavioral disorders and autistic features. Response to AEDs was variable, and most of the patients (more than 50%) presented with frequent seizures despite being treated with several AEDs. A single patient showed a good response after therapy with valproic acid (VPA) and clonazepam, with a reduction of frequency and severity of seizures. Another case of highly drug-resistant epilepsy was treated with three-drug combinations and trials with a ketogenic diet and intravenous methylprednisolone. In some cases, information regarding the response to antiepileptic treatments were not reported (15, 44–47).

## VAMP2

The VAMP2 gene, mapped on chromosome 17 (17p13.1 region), encodes for Vamp2 (also called synaptobrevin-2) (16, 48). As mentioned, Vamp2 protein constitutes the v-SNARE, and results fundamental to driving synaptic transmission, which is also regulated by Ca^2+^ ions and other proteins (16). Pathogenic variants of VAMP2 gene are associated with neurodevelopmental disorders, such as visual impairment, hyperkinetic movements, autism spectrum disorder and epilepsy (16, 39). More severe neurological phenotypes are described in individuals with nonsynonymous mutations of VAMP2 (39). The importance of a correct mechanism of neuronal trafficking mediated by Vamp2 has been highlighted by a recent study evaluating the essential role of *VAMP2* and *DLG4* in the progression of epilepsy and behavioral disorders, in particular ADHD (49). In these conditions, the expression of Dlg4 and Vamp2 is downregulated, which determines abnormal neurotransmission, presumably the cause of these disorders (49).

To date, just three patients carrying pathogenic variants of VAMP2 associated with DEE are described (see Table 3) (16). In particular, Salpietro et al. (16) reported five individuals (from 2 months to 14 years of age) with de novo heterozygous mutations of the VAMP2 gene presenting with various neurodevelopmental phenotypes, such as epilepsy, hypotonia, ID, and autistic features. Two of these patients did not present epileptic manifestations, but they showed EEG anomalies (such as high-voltage delta activity, sharp-and-slow-wave complexes or only a disorganized EEG). Conversely, three patients showed seizures onset within the first months of life: one presented with focal seizures, another one reported generalized tonic-clonic seizures and focal seizures, the last one developed infantile spasms. EEGs showed disorganized activity, generalized and/or multifocal abnormalities, sharp wave-slow wave complexes, or other focal paroxysms. Language is always compromised (1/3) or absent (2/3), and all the patients presented with autistic features and variable motor stereotypies comparable to Rett syndrome (RTT) (3/3) (16). The patients showed highly drug-resistant epilepsy: they trialed several AEDs, such as VPA, vigabatrin, and lamotrigine. VPA has been proved the most beneficial in two individuals, and particularly one of them was reported as seizure-free since the age of 12 years, and his follow-up EEGs were normal (16).

**Table 3:** Clinical features of *VAMP2*-related patients.

**No**.	**Age of onset**	**Seizure type at onset**	**Epilepsy evolution**	**EEG**	**Movement disorders**	**ID/DD**	**Genetic variant**	**Reference**
1	After birth	FS, GTCS	Drug resistant epilepsy	Fast rhytmic activity, sharp wave-slow wave complexes	Generalized chorea	Rett-like	c.233A>C; p.Glu78Ala	(16)
2	1 month	IS	Drug resistant epilepsy-CSE	Disorganized EEG and paroxysms	Choreic movement, myoclonic jerks	Rett-like	c.230T>C; p.Phe77Ser	(16)
3	5 years	FS	ncse	generalized and multifocal abnormalities	Absent	Rett-like	c.128_130delTGG; p.Val43del	(16)

## CPLX1

The *CPLX1* gene is located on chromosome 4 (4p16.3), and it encodes for the complexin 1 (Cplx1). The complexin-family is a group of highly conserved cytosolic proteins expressed at the pre-synaptic terminal and interacting with the SNARE complex: Cplx1 and Cplx2 are the two paralogues mainly expressed in the CNS, the former being the most represented isoform, while Cplx3 and Cplx4 are predominantly identified at retinal ribbon synapses (13, 50, 51).

The exact function of the complexins has yet to be fully unraveled, but two mechanisms of action have been proposed. A controversial role as an inhibitor of the spontaneous release of neurotransmitters has been reported, with stronger evidence in invertebrates than in mammals; in fact, an increase in a spontaneous release of neurotransmitter was observed in cultured complexin-1/2 knockdown cortical neurons but not in complexin-1, −2 and double complexin-1/2 knockdown mice (13, 51–53). Secondly, a role as a Ca2+-triggered release promoter has been suggested, given the evidence showing a reduction in synaptic response amplitude after the action potential stimulation (51). Moreover, a reduction in both evoked and spontaneous release of glutamate is described in complexin-1/2/3 null cultured hippocampal neurons (13, 53). To date, the prominent hypothesis for the complexin mechanism of action is represented by its involvement in regulating the vesicle fusogenicity by lowering the energy barrier for primed vesicles to undergo either Ca2+-evoked or spontaneous fusion (13, 51).

To date, only five patients with DEEs associated with pathogenic variants CPLX1 have been described (50, 54), with epilepsy onset from 6 weeks to a maximum of 2 years of life. Infantile spasms (2/5) were the most frequent seizures type at onset, while a single patient showed myoclonic seizures (50). These three patients (3/5) developed myoclonic seizures, and EEG showed generalized epileptiform activity (50). However, all patients presented drug-resistant epilepsy. Conversely, Karaka et al. (54) reported two sisters that developed malignant migrating epilepsy and unspecified ID (54). No association with autistic features were reported in all the subjects. A single patient showed movement disorders and cerebral palsy (50). Brain MRI resulted normal in two patients (2/5), showed cortical atrophy in two patients (2/5), and in a single case (1/5) detected cerebellar abnormality (50). All data are summarized in Table 4.

**Table 4:** Clinical features of *CPLX1*-related patients.

**No**.	**Age of** **onset**	**Seizure type at onset**	**Epilepsy evolution**	**EEG**	**Movement disorders**	**ID/DD**	**Genetic variant**	**Neuroimaging**	**Reference**
1	NA	NA	Malignant migrating epilepsy	NA	NA	ID	c.322G>T; p.Glu108* (homozygous non-sense variant)	Cortical atrophy	(54)
2	NA	NA	Malignant migrating epilepsy	NA	NA	ID	c.322G>T; p.Glu108* (homozygous non-sense varia-nt)	Cortical atrophy	(54)
3	6 weeks	IS	Migrating myoclonic epilepsy—deceased	Generalized epileptiform activity and hyperexcitability	NA	DD	c.315C>A; p.Cys105* (homozygous non-sense variant)	Small cleft of lobule VIII of the left cerebellar hemisphere with malorientation of the adjacent cerebellar folia (at 1, 2 and 3 years)	(50)
4	2 ½ months	IS	Myoclonic epilepsy (progressive)	Generalized spikes and waves, hyperexcitability	NA	DD	c.315C>A; p.Cys105* (homozygous non-sense variant)	Normal (at 2 months and 2 years)	(50)
5	2 years	Myo	Myo, TS and GTCS	Marked persistent generalized seizure activity	Cerebral movement disorder and cerebral palsy	DD	c.382C>A; p.Leu128Met (homozygous)	Normal (at 17 months)	(50)

## STXBP1

The *STXBP1* (also known as *MUNC18-1*) is a gene located on chromosome 9 (9q34.11 region), which encodes the Syntaxin1a binding protein (Stxbp1), a protein of the SEC1 family that is essential for vesicles trafficking (19, 55). The Stxbp1 is a neural-specific binding protein that organizes the protein complexes that induce secretory vesicle exocytosis (56, 57). Notably, it is crucial to promote the conformation change in Stx1a, allowing the SNARE complex formation (22). This Stxbp1-Stx1a binding serves two purposes: firstly, when Stx1a is in a “closed” conformation, it interacts with the Habc domain and prevents the formation of ectopic and uncontrolled SNARE complexes in the synapses; secondly, when it binds the N-terminal peptide of the “open” Stx1a, Stxbp1 facilitates the synaptic vesicle priming and fusion, allowing the neurotransmitter release (58). It has also been demonstrated that Stxbp1 levels correlate with secretion capacity and synaptic strength, making this protein fundamental in synaptic transmission and maintenance of synaptic connections in adulthood (59–61).

Mutations of the *STXBP1* gene determines an alteration in the elaborate mechanism of synaptic exocytosis, leading to an excitatory/inhibitory imbalance which can trigger an increased epileptic activity (60). Additionally, is ubiquitously expressed in the neuron. Stxbp1 also has non-synaptic functions: it regulates the post-Golgi transport of vesicles, it chaperons the α-synuclein, and it is a fundamental element in the development of the brain allowing neurite extension and radial migration of the cortical neurons (58).

Initially, the *STXBP1* gene was associated with Ohtahara syndrome by Saitsu et al. (62). Since this discovery, the development and wide application of genetic testing helped recognize numerous new *STXBP1*-related severe early-onset DEEs (with a median onset of 6 weeks in 85% of the cases). The epileptic phenotypic spectrum includes West syndrome, Lennox-Gastaut syndrome, Dravet syndrome, early myoclonic encephalopathy, and several unclassified DEEs, with the former and the latter representing together half of *STXBP1*-related epilepsy (58, 63). Moreover, patients with disease-causing variants of *STXBP1* have diverse phenotypes, including Rett-like syndrome and non-epileptic presentations, but they always show severe to profound DD/ID (58, 63, 64). In addition, an evolution of *STXBP1*-associated disease with the development of neurologic symptoms similar to early-onset parkinsonism has been recently pointed out (65).

Given the wide clinical spectrum of *STXBP1*-related diseases, a genotype-phenotype correlation is difficult to achieve: haploinsufficiency of the gene associated with a dominant-negative effect has been proposed as the primary pathogenic mechanism behind *STXBP1* encephalopathy, potentially explaining the complex expression of pathogenic variants of this gene (64, 66). Previous reports showed an association between non-sense mutations and early onset DEEs, while missense pathogenic variants are correlated to more various clinical phenotypes (58). Interestingly, most of the pathogenic variants of *STXBP1* described in the literature are heterozygous mutations, leading to the assumption that they were the only ones tolerated. However, two siblings presenting with Lennox-Gastaut syndrome were recently reported, carrying a homozygous missense mutation of *STXBP1*, causing a gain-of-function (64).

Regarding the treatment options for *STXBP1*-related DEEs, it is noteworthy that the patients carrying a pathogenic variant of *STXBP1* showed seizures refractory to standard AEDs (58). Similarly to most of the neonatal-onset epileptic encephalopathies, Phenobarbital, VPA, vigabatrin, and levetiracetam are the most used drugs, though over half of the patients are treated with more than three AEDs, included ACTH and corticosteroids (58, 63, 67). Novel treatments such as trehalose, sorbitol, and 4-phenylbutyrate have been proven efficacious in restoring Stxbp1 protein levels in primary mouse neurons and *C. elegans* models: these compounds may reverse the deficit caused by the mutant protein and were also able to increase levels of wild-type Stxbp1 (58). Given the above, a clinical trial evaluating the safety and tolerability of Glycerol Phenylbutyrate in a small group of patients began in 2021 and is set to end in 2023 (ClinicalTrials.gov identifier: NCT04937062; accessed on 02 Jan2021).

## SV2A

Synaptic vesicle glycoproteins 2 (Sv2) are a transmembrane glycoprotein family located in the synaptic vesicles of neurons. Three isoforms of Sv2 are reported: Sv2a, 2b and 2c (68). The *SV2A* gene, mapped on chromosome 1 (1q21.2 region), encodes for Sv2a, a protein first described by Buckley and Kelly (69) that consists of 12 transmembrane domains and cytoplasmic N- and C-terminal sequences (70). It is widely expressed in GABAergic and glutamatergic neurons of the cerebral cortex, hippocampus and cerebellum, and it controls synaptic transmission through multiple mechanisms: Sv2a promotes the formation of the SNARE complex; also, it intervenes in both immediate synaptic vesicle release and Ca^2+^-dependent release as it interacts directly with synaptotagmin1 (Syt1). It has also been recognized as an ATP-binding site that would be involved in the process of vesicular priming (71, 72). It also represents the target site of Levetiracetam (LEV) (73). In literature, several studies on animal models showed how *SV2A* missense mutations cause an imbalance in GABAergic and glutamatergic transmission, leading to epilepsy (74–76).

To the best of our knowledge, only three cases of epilepsy associated with mutations in the *SV2A* gene have been reported in humans. Calame et al. (77) described a patient with epilepsy onset at 2 years of age. The administration of LEV caused a worsening of seizures and the development of a status epilepticus. Genetic investigations demonstrated a rare *de novo* variant in heterozygosity in *SV2A*. Subsequently, LEV was suspended, and the patient achieved good seizure control with VPA and a ketogenic diet. Developmental milestones in this patient are described as adequate, ruling out the hypothesis of a DEE.

On the contrary, Wang et al. (78) described a girl with a slight developmental delay and myoclonic seizures. The child was initially treated with levetiracetam, showing a deterioration of the clinical picture with the development of infantile spasms. Nevertheless, epileptic episodes disappeared after the suspension of levetiracetam and the administration of VPA and Clonazepam (78).

Only a case associated with drug-resistant epilepsy and DD/ID was reported by Serajee and Huq (79). This patient showed microcephaly, optic atrophy, epileptic spasms and myoclonus, with onset at 2 months of age (79). The clinical features of these two cases are summarized in Table 5.

**Table 5:** Clinical features of *SV2A*-related patients.

**No**.	**Age of** **onset**	**Seizure type at onset**	**Epilepsy evolution**	**EEG**	**Movement disorders**	**ID/DD**	**Other neurological features**	**Genetic variant**	**Neuroimaging**	**Reference**
1	2 months	IS and arching of the back, Myo	Drug resistant epilepsy—TS	Multifocal spikes, diffuse attenuation superimposed by diffuse fast wave activity; spike and waves	NA	DD	Hypotonia, microcephaly, optic atrophy	c.1148G>A; p.Arg383Gln	Diffuse increased T2 signal in the bilateral frontal, parietal and temporal deep cerebral white matter, thin corpus callosum, and mild ventriculomegaly (at 11 months)	(79)
2	2 months	Myo	Epileptic spasms after LEV administration	Burst of poly-spikes bilaterally	NA	DD	NA	c.1708C>T; p.Arg570Cys	NA	(78)

## PRRT2

The *PRRT2* gene is located on chromosome 16 (16p11.2 region) and encodes for proline-rich transmembrane protein 2 (Prrt2), a pre-synaptic protein widely expressed in the cerebellum, the basal nuclei, and the neocortex (80, 81). Its expression is increased at major synaptogenesis stages, and it intervenes in modulating neuronal excitability. Two mechanisms are responsible for this process: Prrt2 regulates Nav1.2/1.6 currents (82) and contributes to controlling the vesicular trafficking and releasing the neurotransmitters. However, the mechanism of action of *PRRT2* still appears controversial: according to Coleman et al. (83), the N-terminal portion of Prrt2 interacts directly with the SNARE complex reducing its formation, thus causing a decrease in the process of vesicular exocytosis (83). On the contrary, Valente et al. (81) proposed an alternative mechanism of action, according to which Prrt2 participates in the regulation of the Ca^2+^ sensing apparatus for the rapid synchronous release of synaptic vesicles by binding Snap25 and Syt1/2. Specifically, they observed a reduced neurotransmitter release in *PRRT2*-silenced primary neurons (81). Therefore, according to these evidences, in both cases Prrt2 influences the vesicular neurotransmitter release but with two apparently opposite effects (81, 83).

Pathogenic variants of *PRRT2* are associated with a large spectrum of familial neurological disorders: *PRRT2* is primarily associated with paroxysmal dyskinesia (PKD) but also hemiplegic migraine (HM), infantile convulsions and choreoathetosis (ICCA) and benign sporadic and familial seizures (BFIS) (84, 85).

Movement disorders are salient features of *PRRT2*-associated conditions, usually with onset in adulthood, and consisting principally in dystonia and choreoathetosis (84). The PKD chorea and dystonia episodes are typically brief (about 1 min) and can be triggered by prolonged exercise (86).

Regarding epilepsy, BIFS usually occurs within the first year of life and has a good response to drug therapy, especially with carbamazepine, phenobarbital, and VPA (87). Usually, seizures consist of brief episodes of psychomotor arrest, accompanied by generalized hypertonia, cyanosis, and limb jerks. In contrast, ICCA is characterized by the precocious onset of seizures and the subsequent development of movement disorders (paroxysmal dyskinesias), which generally occur at 5 years (86).

A genotype-phenotype correlation of *PRRT2*-related disorders is complicated, as it may be inferred by the wide phenotypic variability of diseases associated with the frameshift mutation c.649dupC (p.R217Pfs^*^8), which is reported as recurrent in the literature (82, 87, 88).

Generally, the neurological outcome is favorable (87), and there are no clear correlations between *PRRT2* and epileptic encephalopathies (89).

Few cases of pathogenic variants of *PRRT2* associated with clinical phenotypes comparable to DEEs were described: Pavone et al. (90) described a single patient with drug-resistant epilepsy, persistent electroencephalographic abnormalities, and severe DD. Djémié et al. (91) and Jafarpour and Desai (92) reported two cases of West Syndrome; Guerrero-López et al. (93) described a patient with epilepsy and severe ID. Moreover, severe conditions have been associated with homozygosity mutations of *PRRT2* (94).

Although *PRRT2* is known to be responsible for cognitive disorders (95), more studies are needed to assess the frequency of ID and psychiatric symptoms in *PRRT2*-associated syndromes (96).

## NAPB

*NAPA* and *NAPB* genes are mapped respectively on 19 (19q13.33 region) and 20 (20p11.21 region) chromosomes, and encoding for the soluble NSF Attachment Proteins Alpha (αSnap) and Beta (βSnap), ubiquitarious proteins with higher expression in the brain, βSnap only being expressed post-natally (97, 98). They are highly homologous to each other and represent essential components in the vesicular transport, the membrane fusion, and the release of neurotransmitters. In particular, they play a crucial role in dissociating and recycling the SNARE complex, making its components available for subsequent fusion reactions (98). The Snap proteins participate as a co-factor of the NSF ATPase during the SNARE complex disassembly, inducing increased levels of the free SNARE components (5, 98).

Given their role in neuronal regulation and brain development, variants of these genes may be associated with various neurological disorders, yet a clear link to developmental and epileptic encephalopathies has not been reported (97). However, a study conducted on βSnap-KOs mice demonstrated an epileptic phenotype with onset 11 days after birth, consistent with the developmental expression pattern of βSNAP: the mice developed severe recurrent epileptic seizures, occasionally leading to death (98).

To the best of our knowledge, solely four patients with DEEs associated with disease-causing variants of *NAPB* are described (29, 97, 99) (see Table 6). Clinical findings of the patient reported by Reuter et al. (29) was unavailable. All the other patients presented a very early onset of seizures (range from 2 to 6 months), and the most frequently reported type of seizures is clonic (3/4), but tonic seizures are also described in association with the clonic ones (97, 99). In the majority of the cases (3/4), the epilepsy evolution is a multifocal epileptic encephalopathy, and all the patients (4/4) presented a severe DD/ID (29, 97, 99). Neuroimaging was reported as normal in 2/4 subjects (2/4 are unavailable) (97, 99). One patient developed movement disorders such as axial and peripheral hypotonia, limb tremulousness and stereotypies (kicking, hand, wrist-twisting, and bringing to the midline) (99). No information about the therapeutic approach was reported.

**Table 6:** Clinical features of *NAPB*-related patients.

**No**.	**Age of** **onset**	**Seizure type at onset**	**Epilepsy evolution**	**EEG**	**Movement disorders**	**ID/DD**	**Other neurological features**	**Genetic variant**	**Neuroimaging**	**Reference**
1	5 months	TS and/or CS	Multifocal epileptic encephalopathy	NA	Limb tremulousness and stereotypies (kicking, hand, wrist twisting, and bringing to the midline)	Profound ID, global DD	Axial and peripheral hypotonia	c.565C>A; p.Ser160*	Normal	(99)
2	6 months	Clonic seizures	Multifocal epileptic encephalopathy	Multifocal epileptic discharges in the C-P-O or F-T regions	NA	Profound ID and global DD	NA	c.433-1G>A (homozygous splicing variant)	Normal	(97)
3	2 months	Clonic seizures	Multifocal epileptic encephalopathy	NA	NA	Global DD, profound ID	Microcephaly	c.433-1G>A (homozygous splicing variant)	NA	(97)
4	NA	NA	Epileptic encephalopathy	NA	NA	Profound ID	Hypotonia, impaired vision	c.173G>A; p.Trp58*	NA	(29)

## DNM1

The dynamin (Dmn) is a GTPase involved in vesicular transport and clathrin-dependent endocytosis (100). There are three different isoforms of the protein, called Dnm 1, 2 and 3; variant 1 is the most expressed in neurons (101). Dnm1 is encoded by the DNM1 gene located in 9q34.11, and its levels increase in parallel with synaptogenesis, particularly during the post-natal phase (102, 103). The process of phosphorylation/dephosphorylation of Dnm1, mediated by Cdk5 and Ca^2+^-dependent calmodulin, respectively, regulates the activation of Dnm1 by facilitating its interaction with other proteins involved in endocytosis. For this reason, pathogenic variants of *DNM1* can cause an impairment of endocytosis of synaptic vesicles with consequent impact on vesicle recycling and synaptic function (104). To date, it has been clarified that DNM1 pathogenic variants can be associated with Lennox-Gastaut syndrome and infantile spasms (105, 106).

Our review of the literature has led to the identification of 33 cases with pathogenic variants of DMN1, but only 30 of these are associated with epilepsy and some degree of ID (100, 103, 106–114). Brereton et al. (115) described some cases presenting a milder phenotype, characterized by autistic symptoms and ID without seizure. Clinical features of patients with DNM1-related DEEs are summarized in Table 7.

**Table 7:** Clinical features of *DNM1*-related patients.

**No**.	**Age of onset**	**Seizure type at onset**	**Epilepsy evolution**	**EEG**	**Movement disorders**	**ID/DD**	**Other neurological features**	**Genetic variant**	**Reference**
1	7 months	Head dropping	NA	Slow spike-wave, hypsarrhythmia	NA	Severe DD	NA	c.443A>G; p.Gln148Arg	(114)
2	11 months	FS	LGS	Hypsarrhythmia	NA	Severe DD	NA	c.127G>A; p.Gly43Ser	(108)
3	10 months	TS, head dropping	Drug resistant CS and AS, Abs	Hypsarrhythmia	Choreic hand movements and distal limb dys- tonia	Severe DD	NA	c.709C>T; p.Arg237Trp	(108)
4	7 months	IS	NA	Slow background, multifocal discharges	Ataxia, mild tremor	Severe ID; NV	Hypotonia	c.529G>C; p.Ala177Pro (*de novo*, missense mutation)	(109)
5	6 months	IS	NA	Hypsarrhythmia	NA	Severe ID	General hypotonia	c.618G>C; p.Lys206Asn (*de novo*, missense mutation)	(109)
6	2 months	IS	NA	Bilateral slow spike-wave	na	Severe ID; NV	General hypotonia	c.1076G>C; p.Gly359Ala (*de novo*, missense mutation)	(109)
7	13 months	IS	NA	Hypsarrhythmia	na	Profound ID	Axial hypotonia	c.194C>A; p.Thr65Asn (*de novo*, missense mutation)	(109)
8	12 months	IS	Myo, Atyp Abs, TS, FS, GTCS, obtundation status	Modified hypsarrhythmia	na	Profound ID	Axial hypotonia	c.709C>T; p.Arg237Trp (*de novo*, missense mutation)	(109)
9	NA	IS	LGS	NA	NA	ID	NA	c.618G>C; p.Lys206Asn	(103)
10	NA	IS	LGS	NA	NA	ID	NA	c.529G>C; p.Ala177Pro	(103)
11	7 months	IS	TS	Multifocal epileptiform discharges, hypsarrhythmia	Dyskinesia; Nystagmus	Profound ID		c.865A>T; p.Ile289Phe	(110)
12	4 months	IS	Myo, GTCS, FS	Bitemporal epileptiform activity	NA	Profound ID	Hypotonia	c.709C>T; p.Arg237Trp	(111)
13	12 months	TS	IS, Myo, GTCS, FS	Bilateral occipital epileptiform activity diffuse background slowing multifocal epileptiform discharges	NA	Profound ID	Hypotonia	c.709C>T; p.Arg237Trp	(111)
14	3 weeks	Myo	Abs	Slow background	NA	Profound ID, NV	Hypotonia	c.127G>A; p.Gly43Ser	(106)
15	8 months	GTCS	NA	Multifocal epileptiform discharges, slow background	NA	Profound ID, NV	Hypotonia	c.731G>A; p.Ser238Ile	(106)
16	3 months	NA	NA	Multifocal epileptiform discharges, slow background activity	NA	Profound ID, NV	Hypotonia	c.1075G>A; p.Gly359Arg	(106)
17	3 months	IS, Myo	Seizure freedom	Hypsarrhythmia, multifocal epileptic discharge	NA	Profound ID, NV	Hypotonia	c.1190G>A; p.Gly397Asp	(106)
18	4 months	IS	Abs, TS, GTCS, SE	Hypsarrhythmia, multifocal epileptic discharge, generalized sharp wave	NA	Profound ID, NV	Hypotonia	c.416G>T; p.Gly139Val	(106)
19	2 months	IS	Abs, TS, Myo	Multifocal epileptiform discharges	NA	Severe ID, NV	Hypotonia	c.616A>G; p.Lys206Glu	(106)
20	6 months	IS	AS, GTCS	Hypsarrhythmia, slow spike-wave discharges, focal epileptiform discharges	NA	Severe ID; NV	Hypotonia	c.709C>T; p.Arg237Trp	(106)
21	3 months	IS	AS, GTCS, FS	Multifocal epileptiform discharges, slow spike-wave, slow background	NA	Profound ID, NV	Hypotonia	c.709C>T; p.Arg237Trp	(106)
22	5 months	IS	AS, Abs, GTCS	Hypsarrhythmia, multifocal epileptic discharge, generalized epileptic discharge, slow background	NA	Profound ID, NV	Hypotonia	c.709C>T; p.Arg237Trp	(106)
23	5 months	IS	Abs, Myo, AS, GTCS	Hypsarrhythmia, multifocal epileptic discharge, generalized spike wave	NA	Profound ID, NV	Hypotonia	c.709C>T; p.Arg237Trp	(106)
24	6 months	IS	Myo, TS	Hypsarrhythmia, multifocal epileptic discharge, focal epileptiform discharge, slow background	NA	Profound ID, NV	Hypotonia	c.1037G>T; p.Gly346Val	(106)
25	1 month	IS	Myo, TS, GTCS, FS, SE	Hypsarrhythmia, multifocal epileptic discharge, slow spike wave, generalized spike wave	NA	Profound ID, NV	Hypotonia	c.1075G>A, p.Gly359Arg	(106)
26	4.5 years	Fs	Myo, TS, GTCS, FS, SE	Generalized spike-wave, slow background	NA	Profound ID, NV	NA	c.1117G>A; p.Glu373Lys	(106)
27	1 day	Myo, TS	IS	Normal activity of background, irregular sharp waves and spike and waves complexes followed by attenuation	NA	Profound ID, NV	Hypotonia	Insertion c.1089_1090inscttcca in exon 8; p.asn363_arg364insleupro	(100)
28	5 days	NA	SE	Diffuse slowing and multifocal epileptiform activity	NA	ID, DD	Hypotonia	c.796C>T; p.Arg266Cys	(112)
29	8 months	IS	IS	Sharpe wave, multifocal epileptic discharge	NA	Severe ID, NV	Hypotonia	c.135C>A; p.Ser45Arg	(107)
30	4 months	GTCS	Abs, FS, GTCS	NA	NA	Severe DD	NA	c.431C>T; p.Pro144Leu	(113)

The onset of seizures occurred in almost all cases within the first year of life, and the most frequently reported seizures type is represented by infantile spasms (19/30). Noteworthy is the case of a patient who did not report further epileptic episodes despite presenting an onset with infantile spasms in the first year of life, developing a neurological impairment with profound ID (106).

All patients presented with severe/profound ID, in many cases (17/30) associated with the absence of verbal communication (100, 106, 107, 109). Also, two-thirds of the cases (22/30) showed deep axial and/or diffuse hypotonia and severe involvement of motor skills (100, 106–109, 111, 112).

It is noteworthy mentioning that the genetic variant c.709C>T (p.Arg237Trp) was found in 8 cases out of 30 (27% of patients identified) and that c.1075G>A (p.Gly359Arg) was reported twice (106, 108, 109, 111).

Data regarding the therapy was not available in 7/30 of the patient. Only four patients (4/30) were seizure-free, and in particular, two of them have achieved the absence of seizures following the ketogenic diet (106, 109, 114). In addition, another one obtained the absence of epileptic episodes for a long time following a ketogenic diet, although epilepsy recurred later (109).

## ZFYVE20

The *ZFYVE20* gene, mapped on chromosome 3 (3p25 region), encodes for Rabenosyn-5 (Rbsn-5), a large, highly conserved, multidomain protein, which is ubiquitously expressed in mammalian cells. Rbsn-5 is involved in receptor-mediated endocytosis and neurotransmitter recycling (116, 117). Specifically, Rbsn-5 main function is to regulate the intracellular route of internalized neurotransmitters receptors, facilitating their recycling to the plasma membrane through direct interaction with regulatory proteins and lipids, such as the endocytic GTPases Rab4 and Rab5, and phosphatidylinositol-3 phosphate (116–118).

We reviewed recent literature and identified a single case in which a pathogenic variant of *ZFYVE20* is associated with a form of DEE (116). A girl with a homozygous missense mutation of *ZFYVE20*, detected by whole-exome sequencing, showed a severe drug-resistant epileptic encephalopathy and ID (116). She presented seizures onset at 5 months of life with infantile spasms and had marked hypotonus, with the impossibility of sitting and walking independently. The patient also presented facial dysmorphisms, microcephaly, macrocytosis and megaloblastoid erythropoiesis (116). She gained poor seizures control with several anticonvulsive drugs (VPA, Phenobarbital, Levetiracetam, Lamotrigine). At 14 months, a ketogenic diet was started with a report of improvement, and she was clinically seizure-free at 6.5 years of age (116).

## TBC1D24

The *TBC1D24* gene, located on chromosome 16 (16p13.3 region), encodes for Tbc1d24, a highly conserved 553 amino acid protein, which consists of two domains, the Tre2/Bub2/Cdc16 domain (TBC) and a TBC/Lysin Motif Domain/Catalytic (TLDc) domain (119). The TBC domain is involved in the regulation of synaptic traffic, while the function of the TLDc domain is less known, but it appears to be involved in oxidative stress processes (119).

Tbc1d24 is involved in vesicle trafficking in the brain, neuronal migration, and somatic cellular development (120, 121). In particular, in the pre-synaptic terminal, it acts as a selective GTPase activating protein for the GTPase Rab35, which allows the endosomal sorting of synaptic vesicle proteins and the replacement of damaged components (122). Moreover, Falace et al. (120) demonstrated that Tbc1d24 binds the GTPase Arf6, proving in mouse models the role of *TBC1D24* in regulating neuronal migration.

Pathogenic variants of this gene are associated with heterogeneous clinical manifestations, including non-syndromic hearing loss and drug-resistant epilepsy, cerebellar alterations, alternating hemiplegia, and symptoms of neurodegeneration (119).

Epilepsy phenotypes associated with pathogenic variants of *TBC1D24* include familial infantile myoclonic epilepsy, epilepsy of infancy with migrating focal seizures (EIMFS) and DOORS (deafness, onychodystrophy, osteodystrophy, intellectual disabilities, and seizures) syndrome (123–125).

In the recent literature, we found the description of 30 cases in which mutations of *TBC1D24* are associated with DEEs (107, 123–131). All clinical features are summarized in Table 8.

**Table 8:** Clinical features of *TBC1D24*-related patients.

**N**.	**Age of** **onset**	**Seizure type at onset**	**Epilepsy evolution**	**EEG**	**Movement disorders**	**ID/DD**	**Other neurological features**	**Genetic variant**	**Neuroimaging**	**Reference**
1	3 months	GTCS, FS	Spasms in the craniofacial region and all of her limbs, as well as a concurrent, sudden decrease in vision manifested as lethargy and constant crying; NCSE	Theta and delta activity in bilateral hemispheres	Cerebellar ataxia	DD		c.1416_1437del/ c.1499C>T; p.Ala500Val	NA	(107)
2	5 weeks	Migrating CS, FS	EIMFS—deceased at 8 years	First interictal: slow background activity, with slow waves, rare paroxysmal activity; interictal stormy phase: multifocal spikes, slow background activity; ictal: focal theta discharge followed by delta large amplitude hemispheric discharge; interictal late phase: absence of any organization, rare spikes in both temporal regions; myoclonic seizures associated with EEG abnormalities (frequency range 0.25-1 Hz)	Dystonic movements	ID	Severe axial hypotonia	c.468C>A; p.Cys156*/c.686T>C (p.Phe229Ser)	moderate brain atrophy sparing the posterior fossa (at 6 months)	(125)
3	4 weeks	Migrating CS, FS	EIMFS—deceased at 18 months	First interictal: slow background activity, with slow waves, rare paroxysmal activity; interictal stormy phase: multifocal spikes low background activity, rare spindles; ictal: focal migrating discharges; interictal late phase: absence of any organization, rare spikes in both temporal regions; myoclonic seizures associated with EEG abnormalities (frequency range 0.25-1 Hz)	NA	DD	Severe hypotonia	c.468C>A; p.Cys156*/c.686T>C (p.Phe229Ser)	One month old: no structural brain abnormality; 9 months old: global brain atrophy (gray matter) sparing the posterior fossa	(125)
4	2 months	CS, Myo	Early-onset epileptic encephalopathy—deceased at 3.5 years	Several waking-sleep EEGs were within normal limits in the early months of the disease; a progressive slowing of the background activity and a gradual regression in the phasic elements of sleep became evident in later records, as periods of waking and sleep became less distinctive, as well as rare and isolated small spikes and multiple spikes that were predominantly in the frontal and central regions	Dystonic episodes (from the second year of life)	ID	NA	chr16:2547714-2547715delGT; p.Ser324Thrfs*3	Diffuse delay in myelination and a thin corpus callosum (at 6 months); diffuse atrophy with dilatation of the cerebral ventricles, subarachnoid space, and brain sulci (at 2 years)	(125)
5	3 weeks	FS, Myo	Early-onset epileptic encephalopathy—deceased at 3.5 years	Monotonous background activity composed of medium voltage and irregular slow waves within theta and delta ranges; amplitude was lower on the right hemisphere	Spastic hemiparesis and dystonia on the left side	ID	NA	chr16:2547714-2547715delGT; p.Ser324Thrfs*3	Diffuse atrophy with right predominance, especially of right hippocampus (at 31 monts); areas of hypoperfusion in right frontal lower and middle, right mesial and lateral temporal, and left mesial temporal areas at brain SPECT	(125)
6	1 months	FS, IS	Early-onset epileptic encephalopathy—deceased at 6.5 years	Early EEGs were reported to have generalized and multifocal multiple spikes as well as spike-waves discharges	NA	ID	NA	chr16:2547714-2547715delGT; p.Ser324Thrfs*3	Progressive, diffuse cerebral and cerebellar atrophy with dilatation of the ventricles, sulci, and subarachnoid space (at 14 and 37 months)	(125)
7	2 months	CS, Myo, FS, GTCS	Early-onset epileptic encephalopathy	Delta rhythm with multifocal paroxysms	NA	ID	NA	c.32A>G; p.Asp11Gly	Brain atrophy (at 8 and 14 months)	(125)
8	2 months	FS, GTCS	Early-onset epileptic encephalopathy	Paroxysmal epileptiform discharges, bouts of intense crying considered ictal on EEG	Choreoatethoid movement, dystonia, spastic quadriplegia	ID	hypotonia	c.731C>T; p.Ala244Val	Elevated glutamine peak (MRI); cerebellar atrophy, volume loss in left frontal lobe, enlargement of temporal horns suggestive of bilateral hippocampal atrophy (CT)	(125)
9	3 months	FS, CS, GTCS	Multifocal	Multifocal independent spike waves (at 13 years)	Ataxia, hand tremor, progressive gait deterioration	Clinical deterioration; NV	NA	c.679C>T; p.Arg227Trp/ c.1544C>T p.Ala515Val	Right hippocampal sclerosis, bilateral cerebellar atrophy, hyperintense signal of the cerebellar cortex (at 9 years)	(125)
10	45 min after birth	Myo, TS, CS, IS	Early-onset epileptic encephalopathy—deceased at 20 months	First interictal EEG recording (on first day of life) unremarkable despite frequent seizures; later ictal EEG showed generalized spike-wave and poly-spike discharges with F-C predominance; progression to burst-suppression before death	Dyskinetic movements with upper limb dystonia	ID	Axial hypotonia	c.1008delT; p.His336Glnfs*12/ c.32A>G; p.Asp11Gly	Normal	(125)
11	20 min after birth	Myo, TS, CS	Early-onset epileptic encephalopathy—deceased at 24 months	First interictal EEG recording (on first day of life) unremarkable despite frequent seizures; later generalized spike-wave and multiple spike-wave discharges with F-C predominance, slowing of the baseline activity and multifocal spikes	Dyskinetic movements with upper limb dystonia	ID	Axial hypotonia	c.1008delT; p.His336Glnfs*12/ c.32A>G; p.Asp11Gly	Prominent fronto-temporal atrophy with widening of the subarachnoid spaces and Sylvian fissures (at 1 month)	(125)
12	1 day	FS, Myo	Early-onset epileptic encephalopathy—deceased at 6 months	NA	NA	ID	NA	c.119G>T; p.Arg40Leu	Normal cranial ultrasound after birth	(125)
13	1 day	FS, IS	Early-onset epileptic encephalopathy—deceased at 10 months	Multifocal interictal epileptiform discharges (sharp waves, fast activity, spikes, polyspikes), disorganized and slow background, between 6 weeks and (at 8 months)	Nystagmoid eye movements	ID	NA	c.1460_1461insA; p.His487Glnfs*71/ c.313T>C; p.Cys105Arg	day 7: normal; day 56: increased T2 signal in left hippocampus, prominent extra-axial cerebrospinal fluid spaces	(125)
14	2 months	Apnea attacks	IS; SRSE	Hypsarrhythmia	Myoclonus	ID	NA	c.442G>A; p.Glu148Lys (maternal segmental UPiD of chromosome 16)	NA	(128)
15	2 weeks	FS	Deceased at 3 months	Multifocal seizure activity	Myoclonus	DD	NA	c.338C>A;p.Ala113Asp/ c.476T>C;p.Leu159Pro	Mild volume loss with minimal progression of myelination	(129)
16	first week	FS	Multiple SE–deceased at 4 years	NA	NA	severe DD	NA	c.338C>A;p.Ala113Asp/ c.476T>C;p.Leu159Pro	Mild brain atrophy	(129)
17	3 weeks	NA	Multiple SE—decease at 1 year	NA	NA	DD	NA	NA (sibling of patients 15 and 16)	NA	(129)
18	3 months	Myo, CS	NA	Slow background activity and rare sharp waves over the C regions of the left hemisphere (from 3 months); numerous spikes over the vertex and the C regions of both hemispheres prevalent on the left side (at 5 years).	NA	DD	NA	c.457G>A; p.Glu153Lys/ c.1142+1G>A	Hypotrophy of the posteroinferior regions of the cerebellum with mild cortical signal hyperintensities, and delayed myelination over the periventricular and temporal regions	(130)
19	15 days	Myo	Multifocal Myo	FAST RHYTHMS OVER THE FRONTAL REGION	NA	DD	NA	c.1499C > T; p.Ala500Val	Cranial magnetic resonance imaging at the age of 5 months revealed prominent sulci, subarachnoid enlargement, and a cavum septum pellucidum.	(131)
20	2 days	FS	Drug resistant epilepsy—SRSE which resulted in her death—deceased at 9.5 months	Increased delta rhythmic activity on the left hemisphere and infrequent multifocal acute waves	NA	DD	NA	c.121C>T; p.Gln41*	NA	(124)
21	2 months	FS	SRSE; drug-resistant epilepsy	Multifocal spikes	NA	DD	NA	c.121C>T; p.Gln41*/c.321T>A; p.Asn107Lys	NA	(124)
22	3 months	FS	Drug-resistant epilepsy	Rhythmic left-T theta activity with evolution to delta activity	NA	DD	NA	c.845C>G; p.Pro282Arg/c.919A>G	NA	(124)
23	3 months	FS	Drug-resistant epilepsy	Interictal diffuse background slowing without epileptiform discharges	NA	DD	NA	c.845C>G; p.Pro282Arg/ c.919A>G	NA	(124)
24	3 months	FS	Myoclonic epilepsy	Diffuse mild background slowing	Fatigue and gait ataxia; Parkinsonism	mIld ID	NA	c.404C>T; p.Pro135Leu/c.1078C>T; p.Arg360Cys	NA	(127)
25	1 month	FS, migrating CS/TS, IS	SE; frequent Myo	Multiple independent ictal foci in different regions	NA	DD	NA	c.404C>T; p.Pro135Leu/c.457G>T; p.Glu153*	NA	(126)
26	2.5 months	NA	EIMFS	NA	NA	DD	NA	c.116C > T; p.Ala39Val/c.1499C > T; p.Ala500Val	NA	(123)
27	1.5 months	NA	EIMFS—deceased at 19 months	NA	NA	DD	NA	c.116C > T; p.Ala39Val/c.1499C > T; p.Ala500Val	NA	(123)
28	8 months	NA	Epileptic encephalopathy	NA	NA	DD	NA	c.116C > T; p.Ala39Val/ c.241_252del; p.Ile81_Lys84del	NA	(123)
29	7 months	NA	Progressive myoclonic epilepsy	NA	NA	DD	NA	c.241_252del; p.Ile81_Lys84del/ c.1153C > T; p.Gln385*	NA	(123)
30	3 months	NA	Progressive myoclonic epilepsy	NA	NA	DD	NA	c.241_252del; p.Ile81_Lys84del/ c.139A > G; p.Ser47Gly	NA	(123)

All patients presented a history of early onset of seizures, ranging from 20 minutes after birth to 8 months of life: in the majority of the patients (28/30), the onset was within 3 months of life, and, among them, 6/30 individuals developed seizures within the first week (107, 123–131).

The most commonly reported type of seizures is migrating focal ones (17/30), followed by migrating clonic ones (9/30), myoclonic ones (9/30), generalized tonic-clonic ones (4/30), epileptic spasm (4/30), tonic ones (3/30), and apnea attacks (1/30) (107, 123–126, 128–131). In six patients, the description of the seizures was not available (123, 129).

All patients developed a drug-resistant epileptic encephalopathy: five patients (5/30) showed epilepsy of infancy with migrating focal seizures (EIMFS) (123, 125, 126). In two cases, an evolution in progressive myoclonic epilepsy has been described, poorly controlled by drug therapy and often triggered by fever (123).

A non-convulsive super-refractory status epilepticus treated with midazolam, ketamine, and pentobarbital was reported in two cases (124). One of these patients was also treated with hypothermia and died at 9 years (124). Another patient presented with super-refractory status epilepticus treated with midazolam or thiamylal (128). Moreover, the patient described by Li et al. (107) presented with non-convulsive status epilepticus treated with intravenous injection of diazepam, while the patient reported by Lozano et al. (129) developed multiple status epilepticus and died at 1 year of age.

Overall, 13 patients (13/30) died at a very young age (ranging from 3 months to 9.5 years of age) (123–125, 129).

The EEGs did not show a typical pattern, and various abnormalities were described: focal epileptiform discharges in different regions of cerebral hemispheres were reported in seven cases (7/30) (124–126), while 8/30 patients showed generalized or and/or multifocal discharges (125). A diffuse background slowing was described in nine patients (9/30), despite the presence or absence of focal/multifocal discharges (124, 125, 127), while 3/30 initially presented EEGs within the normal limits (125). Lately, 5/30 patients showed a total absence of organization (125). One patient (1/33) developed a burst-suppression pattern (125) and one (1/30) presented with hypsarrhythmia at 4 months of age (128). In 8/30 cases, EEG data was not available (123, 125, 129).

All of them showed DD/ID, and in many cases, the expressive language was inadequate or dysarthric (107, 123–126, 128–131).

In one case, a young woman with an early onset of myoclonic epilepsy showed movement disorders (1/30) with cerebellar ataxia and fatigue, parkinsonism and symptoms of psychosis with hallucinations and depression (127). Other reported movement disorders were Dystonic movements (6/30), cerebellar ataxia (2/30), dyskinetic movements (2/30), non-epileptic myoclonus (2/30), spastic hemiparesis (1/30), choreoatethoid movements (1/30), spastic quadriplegia (1/30), nystagmoid eye movements (1/30) (107, 124–126, 128, 129). In 7/30 cases, the description of movement disorders and other additional features was not available (123, 130, 131).

Most of the patients (29/30) developed an epileptic encephalopathy with frequent seizures non-responsive to various therapeutic approaches and ketogenic diet therapy (107, 123–125, 128–131). Fang et al. (126) described a single patient that experienced >50 % seizure reduction after diazepam treatment. The ketogenic diet was adopted in two cases (123, 124), causing a reduction of seizures and cognitive improvement after a year and a half of diet in one patient (123). In one case, a description of the therapeutic response was not available (127).

## Discussion

DEEs are a heterogeneous group of conditions with onset in infancy or early childhood, in which the epileptic activity significantly interferes with the development, determining severe DD/ID and other neuropsychiatric disorders (9, 10). These diseases may be caused by an alteration of the synapse, the fundamental unit of signal transmission in the nervous system (1, 3). Numerous genes contribute to the synaptic transmission's proper functioning, and alterations of this complex mechanism may result in synaptopathy (1, 8).

We reviewed the literature, focusing on those genes involved in the correct operation of the pre-synaptic terminal, and analyzed the clinical features of 119 patients that showed a clinical presentation resembling a DEE.

A valid genotype-phenotype correlation is difficult to deduce especially in those reports including only few patients with DEEs (50, 79, 116). On the contrary, when many patients with pathogenic variants of the same gene present with different phenotypes, a clear correlation is difficult to achieve: this is even truer in those cases in which the same mutation is related to various clinical presentations (58, 63, 64, 82, 87, 88). This phenotypic heterogeneity has been long studied, and it is related to several factors intervening during the development, including epigenetic factors, timing and location of physiological gene expression and modifier genes (9).

Nevertheless, some noteworthy features may be underlined regarding the clinical phenotypes of DEEs related to the genes we reviewed, which can lead the clinician to a genetic suspect.

For instance, patients with *VAMP2* pathogenic variants showed a neurodevelopmental disorder characterized by ID, central visual impairment, movement disorders, epilepsy or electroencephalographic abnormalities, autistic features, and loss of purposeful hand movements resembling Rett syndrome (16).

STX1B-related DEE must be hypothesized when a patient presents with myoclonic seizures at onset (35, 40–42). Subsequently, these patients may manifest ataxia (35, 37, 39).

Patients carrying *DNM1* mutations may present with infantile spasms in more than a half of the cases, usually associated with axial and/or diffuse hypotonia with severe impairment of motor skills (100, 106–109, 111, 112).

Almost the totality of the patients with a pathogenic variant of TBC1D24 present the first seizure within 3 months after birth, with severe progression frequently leading to death within the first decade of life (107, 123–131). Although EIMFS was reported as a typical epileptic phenotype related to *TBC1D24*, in our review, we could identify 5/30 (16%) patients that developed this type of DEE (123–126). Instead, noteworthy mentioning is the association with status epilepticus (107, 124, 128, 129).

Usually, patients with *SNAP25*-DEEs show seizure's onset after 2 years of age, with generalized seizures and a frequent association to movement disorders (15, 44–47), while *NAPB*-associated DEEs are characterized by a high frequency of clonic seizures and an evolution in multifocal epileptic encephalopathy (97, 99).

Overall, when a patient presents with some degree of DD, an early epilepsy onset with drug-resistant seizures, possibly associated with a movement disorder, the suspect of a synaptopathy must be taken into account when approaching the differential diagnosis.

Concerning the therapeutic approaches, an individually-tailored treatment is desirable to intervene directly on the altered mechanism determining the DEE, improving the seizure control and the developmental outcome. However, in most severe epilepsies, a gene-specific therapy is not available, and the treatment options are represented by the usual AEDs, that do not address the underlying causative mechanism (9). Our literature review regarding DEEs related to genes involved in pre-synaptic mechanisms confirms these data: most patients showed a bad prognosis with highly drug-resistant seizures, despite the multiple therapeutic combinations. Moreover, literature data suggested the efficacy of the ketogenic diet in part of the patients with DEEs related to *DNM1, TBC1D24*, and *ZFYVE2O*. In particular, two subjects with pathogenic variants of *DNM1* achieved the absence of seizures, and another one obtained the absence of epileptic episodes for a long time, although epilepsy recurred later (106, 109). The ketogenic diet was also considered as a valid therapeutic approach for seizure control in two cases related to *TBC1D24*, and especially one of these patients showed a decrease in the frequency of seizures and cognitive improvement after a year and a half of diet (123, 124). Finally, the girl with a disease-causing variant of *ZFYVE2O*, after several AEDs trials, started a ketogenic diet, obtaining a gradual improvement and seizure freedom at 6.5 years of age (116).

This work's limitation is the paucity of a complete description of the patients, making it difficult to obtain homogenous information and, therefore, to deduce a clear genotype-phenotype correlation. A more detailed clinical description of the patients may be desirable to improve the genotype-phenotype correlation and better guide the choice of the genetic testing, allowing to obtain an early diagnosis and to develop individually-tailored therapies.

## Author Contributions

AN and GD conceived planned and supervised the study. GS, GV, and MS wrote the first draft of the manuscript and prepared the tables. GS prepared the figure. AB, GA, and VS helped supervise the project. All authors contributed to manuscript revision, read, and approved the submitted version.

## Conflict of Interest

The authors declare that the research was conducted in the absence of any commercial or financial relationships that could be construed as a potential conflict of interest.

## Publisher's Note

All claims expressed in this article are solely those of the authors and do not necessarily represent those of their affiliated organizations, or those of the publisher, the editors and the reviewers. Any product that may be evaluated in this article, or claim that may be made by its manufacturer, is not guaranteed or endorsed by the publisher.

## Abbreviations

Abs: Absence
AS: atonic seizure
Atyp Abs: Atypical Absence
CS: clonic seizures
CSE: convulsive status epilepticus
DD: developmental delay
EIMFS: Epilepsy of Infancy with Migrating Focal Seizures
FS: Focal Seizure
GTCS: generalized tonic clonic seizure
ID: intellectual disability
IS: Infantile Spasm
LGS: Lennox Gastaut Syndrome
MA: myoclonic atonic seizure
MAE: myclonic atonic epilepsy
Myo: Myclonic seizure
NA: not available
NCSE: non-convulsive status epilepticus
NV: non-verbal
SE: Status Epilepticus
TS: Tonic Seizure.
